# Design and Control of an Upper Limb Bionic Exoskeleton Rehabilitation Device Based on Tensegrity Structure

**DOI:** 10.1155/2024/5905225

**Published:** 2024-08-29

**Authors:** Peng Ni, Jianwei Sun, Jialin Dong

**Affiliations:** ^1^ School of Applied Technology Changchun University of Technology, Changchun 130012, China; ^2^ School of Electrical Engineering Changchun University of Technology, Changchun 130012, China

## Abstract

Upper limb exoskeleton rehabilitation devices can improve the quality of rehabilitation and relieve the pressure of rehabilitation medical treatment, which is a research hotspot in the field of medical robots. Aiming at the problems such as large volume, high cost, low comfort, and difficulty in promotion of traditional exoskeleton rehabilitation devices, and considering the lightweight, discontinuous, high flexibility, and high biomimetic characteristics of tensegrity structure, we designed an upper limb bionic exoskeleton rehabilitation device based on tensegrity structure. First, this article uses mapping methods to establish a mapping model for upper limb exoskeletons based on the tensegrity structure and designs the overall structure of upper limb exoskeletons based on the mapping model. Second, a bionic elbow joint device based on gear and rack was designed, and the stability of the bionic elbow joint was proved using the positive definite matrix method. This device can simulate the micro displacement between bones of the human elbow joint, improve the axial matching ability between the upper limbs and the rehabilitation device, and enhance the comfort of rehabilitation. Third, an impedance control scheme based on back propagation (BP) neural network was designed to address the low control accuracy of flexible structures and patient spasms. Finally, we designed the impedance control scheme of the PSO–BP neural network based on a fuzzy rehabilitation state evaluator. The experimental results show that the exoskeleton rehabilitation device has good flexion motion stability and assist ability and has significant advantages in volume and mobility. The control strategy proposed in this paper has high control precision and adaptive ability and has potential application value in the field of medical rehabilitation.

## 1. Introduction

At present, more than 100 countries and regions around the world are entering the stage of population aging, the global aging is expected to reach 30% by 2050. With this, the phenomenon of “difficulty in accessing medical care and shortage of rehabilitation medical resources” is becoming more and more obvious [1].

The upper limb exoskeleton rehabilitation equipment, using reasonable bone structure and stable control strategy [2], can better simulate the active and passive rehabilitation training process of patients in the rehabilitation process and has become the key research object of medical institutions and relevant scholars at home and abroad. It has become an effective way to relieve the pressure of doctors and patients, improve the quality of life of patients, and ensure the stable development of the rehabilitation medical industry. Many scientific research institutions and medical institutions continue to carry out research on the upper limb exoskeleton rehabilitation device [3, 4]. In 1960, the United States General company designed the first exoskeleton Hardiman because the relevant technology was relatively backward at that time, the exoskeleton structure was very complex, and the whole machine weight reached 680 kg, so this exoskeleton was not promoted. In 2007, the University of Washington designed the seven degree of freedom exoskeleton robot CADEN-7, which was applied to the rehabilitation of patients' shoulders and elbows. Because this robot has more freedom, the overall volume of the robot is larger, and the auxiliary device is more complex, and the production cost is high. In 2010, Nanyang Technological University designed the exoskeleton NUWA, a robot that transfers most of the gravity of the exoskeleton to the human back. This exoskeleton reduces the overall weight of the robot by optimizing the structure, but the total weight is still 10 kg. The University of Texas designed HARMONY, a two-arm exoskeleton. The robot considers the movement law of human joints and realizes the rehabilitation movement of human shoulders and elbows. Due to the large size and high cost of robots, they are mainly used in scientific research. The ReWalk exoskeleton robot, designed by Israeli company ReWalk Robotics, uses a spring-assisted approach to reduce the weight at the end of the device and reduce the complexity of the operating system. The ReWalk weighs 20 kg and can run for 160 min.

Experiments have proved that exoskeleton has high auxiliary capacity and load-bearing capacity, which can effectively improve rehabilitation efficiency and rehabilitation quality [5, 6]. However, due to the rigid structure of exoskeleton brings the problems of large volume, large mass, complex structure, and high cost, which greatly limits its applicability and popularization. Most of the rigid exoskeletons are in rigid contact with the body, and motion is accomplished by applying pressure and friction to the point of contact. This usually generates large forces at the point of contact, reducing the comfort of wearing the exoskeleton, andcreates many safety hazards in the rehabilitation process. Compared to the rigid structure, the flexible structure exhibits characteristics such as small inertia and a simple connection structure. It also demonstrates superior performance in terms of volume, energy consumption, and interaction security of the exoskeleton.

In 2011, Park et al. [7] designed Active Soft Orthotic, a flexible ankle exoskeleton. The exoskeleton is made of flexible materials that provide power without limiting the freedom of the ankle joint. Coincidentally, in 2011, Kenneth Holt and others from Harvard University designed a flexible lower limb exoskeleton robot. The flexible lower limb exoskeleton uses pneumatic muscles as the power source, which strongly proves that the flexible exoskeleton can effectively reduce the metabolic level of the human body during walking. In 2017, Binh Khanh Dinh, Michele Xiloyannis, Leonardo Cappello, and others jointly designed an upper limb flexible exoskeleton robot. This upper limb exoskeleton robot performs excellently in flexibility, comfort, and safety through two elastomers.

Experiments have proved that rehabilitation devices based on flexible structures have shown excellent performance in terms of volume, energy consumption, cost, and mobility. However, many traditional flexible structures are constructed using flexible materials, which fail to address the inherent shortcomings of rigid structures. Traditional flexible exoskeletons need to be further improved in terms of volume, mass, and mobility. Especially in the field of rehabilitation, the comfort and safety issues of traditional flexible structures deserve our attention [8]. In particular, the low degree of matching between the rotational structure of traditional elbow joints and the structure of human elbow joints poses a safety hazard, which deserves continuous attention from experts.

In 1962, the famous American architect Buckminster Fuller proposed the concept of tensegrity structure [9]. Tensegrity structure, which is highly flexible, self-stabilizing, and lightweight, shows great potential in the field of architecture. With scholars from various countries exploring and researching the tensile tensegrity structure, the structure has received great attention from experts in the field of robotics. Kimber et al. [10] developed a modular soft robotic vibration platform based on a triclinic tensegrity structure. Friesen team members at the University of California have proposed robots capable of climbing pipes based on tetrahedral tensegrity structure [11, 12]. Bohm et al. [13, 14] developed a spherical tensioning monolithic robot based on bent rod members.

In order to solve the above key technical problems, taking into account the lightweight, discontinuous, high flexibility, and high biomimetic characteristics of the tensegrity structure, this paper proposes an upper limb bionic exoskeleton rehabilitation device based on tensegrity structure. First, this paper takes the human upper limb as the research object; analyzes the structural characteristics and movement rules of the human upper limb from three aspects of physiology, kinesiology, and rehabilitation; determines the bionic structure simplification scheme and the equivalent replacement principle; and establishes the upper limb skeleton and muscle system simplification diagram. Second, using the characteristics of the tensioned integral structure, and taking the two-bar four-cable tensioned integral structure as the research object, the bionic elbow joint mapping model based on the tensioned integral structure is established [15], and the stability of the bionic elbow joint is analyzed. Third, the bionic elbow joint structure is optimized by adding a rotating pair structure device and a rotating structure device based on gear and rack meshing. Fourth, this paper completed the structural design of the upper arm, elbow joint, and forearm of the upper limb exoskeleton rehabilitation device.

This article designs an impedance control scheme based on back propagation (BP) neural network to address issues such as low precision in flexible structure control and the impact of spasms on control system. Then, based on fully considering the experience of artificial rehabilitation, a fuzzy rehabilitation state evaluator was designed to address the nonlinear characteristics of stiffness and damping. The convergence speed and local optimization problem of the BP neural network were optimized using particle swarm optimization (PSO) algorithm. Finally, we designed the impedance control scheme of the PSO–BP neural network based on a fuzzy rehabilitation state evaluator, which not only improves control accuracy but also enhances the adaptability and safety of rehabilitation exoskeletons.

The main innovations of this paper are as follows:

1. Aiming at the problems of traditional exoskeletons such as large size, high cost, and not easy to be popularized, this paper proposes an upper limb exoskeleton device based on a tensegrity structure.
2. Aiming at the problem of low degree of matching between the traditional elbow joint structure and the human elbow joint, this paper proposes an elbow joint rotation device based on gear and rack. The elbow joint rotation device based on gear and rack mesh is used to compensate the axis offset of elbow joint movement, improve the bionic ability of elbow joint and the rehabilitation comfort.
3. Considering the complexity of rehabilitation movement, this paper designs a fuzzy rehabilitation state evaluator. The fuzzy rehabilitation state evaluator is used to fully summarize the experience of artificial rehabilitation, analyze the rehabilitation state of patients in real time, and improve the safety of the rehabilitation device.
4. BP neural network impedance parameter controller based on PSO is designed. The PSO algorithm is used to optimize the convergence speed and optimization performance of BP neural network, which improves the adaptive self-adaptability of the rehabilitation device.

The rest of this article is organized as follows: Section 2 introduces the structural design of an upper limb exoskeleton rehabilitation device based on the tensegrity structure.  Section 3 introduces the control strategy based on a fuzzy rehabilitation state evaluator [16, 17].  Section 4 introduces the test verification. Finally, Section 5 summarizes this paper.

## 2. Structural Design

### 2.1. Mechanical Mapping and Bionic Shape Finding of the Upper Limb

**Figure 1:**
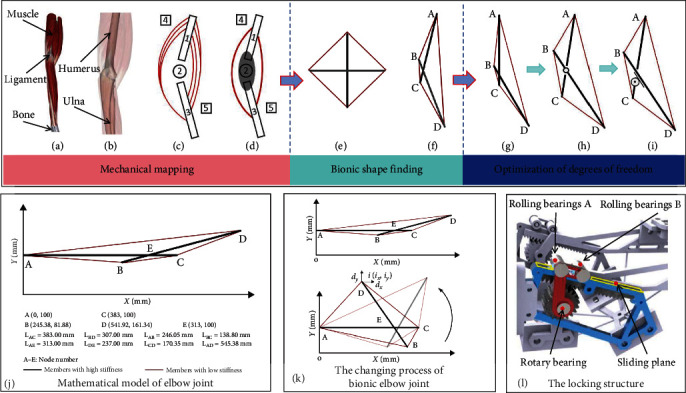
Bionic elbow joint. (a–d) Mechanical mapping of upper limb. (e and f) Bionic elbow joint mapping mode based on tensegrity structure. (g–i) Structural optimization of bionic elbow joint. (j and k) Stability analysis of bionic elbow joint structure. (l) The locking structure of bionic elbow joint.

The upper limb is the part of the body structure with the widest range of motion and the greatest rotation [18, 19]. The human upper limb is composed of muscle tissue [20], ligament tissue, and bone tissue, and its physiological structure is shown in Figure 1(a). The bone structure of the upper limb is mainly composed of humerus, radius, ulna, and elbow joint, and its physiological structure is shown in Figure 1(b). In the process of human upper limb movement, the bones are responsible for rigid support, while the muscles and ligaments are responsible for flexible drive [21]. Ligaments assist the smooth movement of the elbow joint and limit the degree of freedom.

Analyze the structure characteristics and movement rules of human upper limbs, and determine the bionic structure simplification scheme and equivalent replacement principle. According to the motion characteristics of bones during the movement of human upper limbs, it establish the upper limb musculoskeletal relationship diagram, as shown in Figure 1(c). The humerus with small shape variable is simplified as 1 in Figure 1(c), and the elbow joint structure is simplified as revolute pairs 2 in Figure 1(c). Because of the small relative displacement of the radius and ulna, the radius and ulna are simplified as 3 in Figure 1(c). Straightening of the upper limb is mainly completed by the triceps brachii. The upper end of triceps brachii is connected with three points such as scapula, and the lower end is combined into one point, which is simplified as 4 in Figure 1(c). The flexion of the upper limb is mainly completed through the coordination of the brachial muscle, the brachioradialis muscle and the biceps brachii, which is simplified as 5 in Figure 1(c). Combine the same acting muscles to create a simplified diagram of the skeletal and muscular system of the upper limb, as shown in Figure 1(d). The gray ellipse in Figure 1(d) is a simplified diagram of the elbow joint system.

The tensegrity structure consists of a group of continuous tension elements and a group of discontinuous compression elements. Due to the characteristics of self-stability, self-adaptability, self-recovery, deformation, and flexibility, it has become the focus of many experts in the field of mechanical structure design [22]. Rieffe, Scarr and other well-known scholars have proved through research that, starting from the biological characteristics, the tensegrity structure is highly similar to human cell structure, soft tissue structure, and bone structure and has high research value and application value in the field of bionic structure.

The research object of this topic is the tensegrity structure with two bars and four cables, as shown in Figure 1(e). According to the deformation characteristics of tensegrity structure, the whole shape can be changed by changing the length of rigid compression element and flexible tension element. According to the principle of bionic replacement, rigid rods are used to replace the bones with small structural variables, and flexible cables are used to replace the muscles and ligaments that drive the upper limb structures. The two-bar tensegrity structure has the same structural characteristics as the simplified diagram of upper limb skeleton and muscle system. The deformation process of the tensegrity structure is very similar to the motion process of the elbow joint. Analyze the motion mechanism of the elbow joint, and establish a bionic elbow joint mapping model based on the characteristics of the tensegrity structure, as shown in Figure 1(f). The human humerus 1 in Figure 1(d) is mapped to the rigid bar AC in Figure 1(f), and the radius and ulna 3 in Figure 1(d) are mapped to the rigid bar BD in Figure 1(f). The upper limb straightening action muscle 4 in Figure 1(d) is mapped to the flexible elastic members AB and CD in Figure 1(f), and the upper limb flexion action muscle 5 in Figure 1(d) is mapped to the flexible elastic members AD and BC in Figure 1(f).

### 2.2. Stability Analysis of Bionic Elbow Joint Structure

The stability analysis of bionic elbow joint structure is mainly completed through the stability analysis of the bionic elbow joint mapping model based on the tensegrity structure in the previous section. Considering the age distribution of patients, the upper limb size of middle-aged males was selected as the structural parameter of the bionic elbow joint. According to the human dimensions of chinese adults (GB/T 10000-2023), middle-aged men in a relaxed state have a forearm length of 237 mm, an upper arm length of 313 mm, and a default elbow joint angle of 5–15°. The upper limb exoskeleton structure proposed in this article is composed of flexible tensegrity units, which have the deformation characteristics of a tensegrity structure and improve the adaptive ability of the exoskeleton.

According to the size parameters of human upper limb structure, the mathematical model of bionic elbow joint based on coordinate system is established as shown in Figure 1(j). AC represents the upper arm, BD represents the forearm, E represents the initial motion state of the elbow joint, and the motion state of E changes with the deformation of the tensegrity structure.

The stability of the bionic elbow joint structure can be judged by calculating the positive definiteness of the tangent stiffness matrix *K* of the mathematical model of the bionic elbow joint based on the tensegrity structure. If the structure is in the position of minimum potential energy and the stiffness matrix *K* is positive definite, the structure will be in a stable state [23, 24]. If the tangent stiffness matrix *K* is positive, the bionic elbow joint structure is stable, otherwise, the structure is unstable.

The stiffness matrix *K* of bionic elbow joint structure is equal to the sum of elastic stiffness matrix *K*^E^ and geometric stiffness matrix *K*^G^, and the stiffness *K* matrix is:

(1) $$\begin{array}{c} \begin{array}{c} K=K^{E}+K^{G} \end{array}. \end{array}$$

By judging the positive definiteness of *K*^E^ and *K*^G^, determine the positive definiteness of *K*, and finally determine the stability of the bionic elbow joint. Since the two tensegrity structures belong to the category of super stable structures, and the tensegrity structure has nonzero displacement *d*, *K*^G^*d* ≥ 0, the geometric stiffness matrix *K* is a semipositive definite matrix, that is, *K*^G^ is nonnegative (*K*^G^*d* ≥ 0). The stability of bionic elbow joint structure can be inferred by judging the positive determination of elastic stiffness matrix *K*^E^, and the elastic stiffness matrix *K*^E^ is:

(2) $$\begin{array}{c} K^{E}=\left(FL^{-1}\right)G{\left(FL^{-1}\right)}^{T}. \end{array}$$

In Equation (2), *F* is the node equilibrium matrix of the bionic elbow joint tensegrity structure, *L* is the diagonal matrix of the initial length of the bionic elbow joint tensegrity structure member, and *G* is the diagonal matrix of the stiffness form variable of the bionic elbow joint tensegrity structure member. Through calculation, *F*,  *L*, and *G* are introduced into Equation (2) to judge the positive definiteness of elastic stiffness matrix *K*^E^.

The diagonal matrix *L* of the initial length of the bionic elbow joint tensegrity structure member is established and calculated according to Figure 1(j). The four nodes of the bionic elbow joint tensegrity structure are A, B, C, and D. The six constituent units of this structure are AB, AD, CD, BC, AC, and BD. The corresponding relationship of rigid element length is defined as follows: *L*_AC_=|AC| and *L*_BD_=|BD|. The corresponding relationship of elastic element length is defined as follows: *L*_AB_=|AB|, *L*_BC_=|BC|, *L*_**CD**_=|CD| and *L*_AD_=|AD|. Take the length of each element into formula *L*=diag(*L*_AC_, *L*_BD_, *L*_AB_, *L*_BC_, *L*_CD_, *L*_AD_) to calculate the diagonal matrix *L*, the diagonal matrix is:

(3) $$\begin{array}{c} L=\text{diag}\left(383,307,545.38,246.05,138.8,170.35\right). \end{array}$$

Definition *d*_*x*_, *d*_*y*_(∈*R*^4^) is the coordinate vector of node *i*(*i*_*x*_, *i*_*y*_ ) in the *x* and *y* directions. Under the action of external force, the position and length change process of four nodes and six elements of bionic elbow joint is shown in Figure 1(k).

Define *k*_*i*_ as the ratio of force and length change on the *i*(*i*=1, 2, 3, 4, 5, 6) element, and the force density coefficient matrix of bionic elbow joint tensegrity structure is established as *k*=(*k*_1_,  *k*_2_, *k*_3_ ⋯ *k*_6_) ∈ *R*^6^. The force density matrix of the bionic elbow joint tensegrity structure is obtained as *Q* ∈ *R*^(6 × 6)^, and the force density matrix is:

(4) $$\begin{array}{c} Q=\text{diag}\left(k\right). \end{array}$$

Establish the geometric topology matrix C of the bionic elbow joint tensegrity structure. Extract the coordinate vectors of nodes A, B, C, and D of the tensegrity structure to obtain the coordinate matrix *C*_*x*_ and *C*_*y*_ in the *x* and *y* directions, and the coordinates of nodes as shown in Table 1.

The balance equations of all nodes of the bionic elbow joint tensegrity structure in directions *x* and *y* are:

(5) $$\begin{array}{c} C^{T}QC_{x}=d_{x}, \end{array}$$

(6) $$\begin{array}{c} C^{T}QC_{y}=d_{y}. \end{array}$$

Create matrix *E* ∈ *R*^(4 × 4)^ as:

(7) $$\begin{array}{c} E=C^{T}QC. \end{array}$$

Because of the deformation characteristics of the tensegrity structure, the deformation of the tensegrity structure of the bionic elbow joint is determined by the position coordinates of each node. Equations (5), (6), and (7) can be written as:

(8) $$\begin{array}{c} E_{x}=d_{x}, \end{array}$$

(9) $$\begin{array}{c} E_{y}=d_{y}. \end{array}$$

Combine Equations (8) and (9) to get Equation (10).

(10) $$\begin{array}{c} E\left[x\;y\right]=C^{T}QC\left[x\;y\right]=\left[d_{x}\;d_{y}\right], \end{array}$$

where [*x* *y*] is the node coordinate matrix of the elbow joint tensegrity structure. Equation (4) is introduced into Equation (10), and the node equilibrium equation of bionic elbow joint tensegrity structure is obtained by combining and sorting out as follows:

(11) $$\begin{array}{c} Fk=\left(\begin{array}{c} C^{T}\text{diag}\left(C_{x}\right)\\ C^{T}\text{diag}\left(C_{y}\right) \end{array}\right)k=\left[d_{x}\;d_{y}\right]. \end{array}$$

*F* is the node balance matrix of the bionic elbow joint tensegrity structure.

In the elbow joint tensegrity structure, each component is connected to the node, so the component vector can be determined by the node. The vector connection relationship of bionic elbow joint tensegrity structure components is shown in Table 2.

The node vector correlation matrix of the bionic elbow joint is as follows:

(12) $$\begin{array}{c} C=\left[\begin{array}{cccc} 1 & -1 & 0 & 0\\ 1 & 0 & 0 & -1\\ 0 & 0 & 1 & -1\\ 0 & 1 & -1 & 0\\ 1 & 0 & -1 & 0\\ 0 & 1 & 0 & -1 \end{array}\right]. \end{array}$$

Bring the vector incidence matrix *C* and force density matrix *Q* into Equation (7), the matrix *E* is:

(13) $$\begin{array}{c} E=C^{T}QC. \end{array}$$

*Q*=diag(*k*)=diag(*k*_1_, *k*_2_, *k*_3_, *k*_4_, *k*_5_, *k*_6_) in matrix *E*. After calculation and collation, the matrix *E* is:

(14) $$\begin{array}{c} E=\left[\begin{array}{cccc} k_{1}+k_{2}+k_{5} & -k_{1} & -k_{5} & -k_{2}\\ -k_{1} & k_{1}+k_{4}+k_{6} & -k_{4} & -k_{6}\\ -k_{5} & -k_{4} & k_{3}+k_{4}+k_{5} & -k_{3}\\ -k_{2} & -k_{6} & -k_{3} & k_{2}+k_{3}+k_{6} \end{array}\right]. \end{array}$$

According to the structural characteristics of the elbow joint, ignoring the external force and gravity, and according to Equation (10), the equation can be deduced as follows:

(15) $$\begin{array}{c} E\left[xy\right]=\left[00\right]. \end{array}$$

Bringing Equation (14) into Equation (15), the equation can be deduced as follows:

(16) $$\begin{array}{c} E\left[\begin{array}{cc} 0 & 100\\ 383 & 100\\ 245.38 & 81.88\\ 541.92 & 161.34 \end{array}\right]=\left[\begin{array}{cc} 0 & 0\\ 0 & 0\\ 0 & 0\\ 0 & 0 \end{array}\right]. \end{array}$$

After calculation, *k*_1_=−0.801,  *k*_2_=0.223, *k*_3_=−0.944, *k*_4_=3.385, *k*_5_=0.757, *k*_6_=1. Force density matrix *Q*=diag(*k*)=diag(−0.801, 0.223, −0.944, 3.385, 0.757, 1).

Analyzing the state of force density, according to *k*_2_, *k*_4_, *k*_5_, *k*_6_ > 0 and *k*_1_, *k*_3_ < 0, it is judged that the geometric model of the bionic elbow joint tensegrity structure has a good relationship with the structural parameters.

In the bionic elbow joint tensioned monolithic structure, the stiffness deformation variable of the spring elements is the force density of the spring unit. Because the bar element is a rigid structure, the deformation can be ignored, and the stiffness deformation variable is 0. The diagonal matrix *G* of the stiffness form variable of the bionic elbow joint tensegrity structure member is:

(17) $$\begin{array}{c} G=\text{diag}\left(0,k_{2},0,k_{4},k_{5},k_{6}\right). \end{array}$$

Bringing *K*_*i*_ into Equation (17) is:

(18) $$\begin{array}{c} G=\text{diag}\left(0,0.233,0,3.385,0.757,1\right). \end{array}$$

The node balance matrix *F* of the bionic elbow joint tensegrity structure is:

(19) $$\begin{array}{c} F=\left[\begin{array}{c} C^{T}\text{diag}\left(C_{x}\right)\\ C^{T}\text{diag}\left(C_{y}\right) \end{array}\right]. \end{array}$$

Bringing Equations (3), (18), and (19) into Equation (2). Use MATLAB to calculate the eigenvalue *K*^*E*^ as:

(20) $$\begin{array}{c} \text{eig}\left(E^{K}\right)=\left[5.8967,2.6506,1.7201,0,0.0523,0,0,0\right]. \end{array}$$

Because the quadratic form *Q*(*K* ^G^) > 0 of *K*^E^, *K*^E^ is determined to be a positive definite matrix, so *Q*(*K*)=*Q*(*K*^E^+*K* ^G^)=*Q*(*K*^E^ )+*Q*(*K* ^G^) > 0. Finally, the stiffness matrix *K* > 0 is obtained, and *K* is a positive definite matrix, which proves that the bionic elbow joint tensegrity structure has stability and is suitable for the wearable upper limb rehabilitation exoskeleton structure.

### 2.3. Optimization of Bionic Elbow Joint Structure

This chapter mainly focuses on the bottleneck phenomena such as poor comfort of upper limb exoskeleton rehabilitation device. Due to the excessive freedom of the tensegrity structure, the exoskeleton structure still has problems such as poor rigidity and potential safety hazards [25]. Aiming at the stability and safety problems caused by too much freedom of bionic elbow joint based on tensegrity structure. In this paper, a rotating joint structure is added to limit the redundant degrees of freedom of the bionic elbow joint in actual motion, and improve the stability and safety of the bionic elbow joint [26].

Analyze the motion mechanism of the bionic elbow joint model based on the tensegrity structure, as shown in Figure 1(g). On the premise that the rotation structure of the bionic elbow joint remain unchanged, a rotation pair is added at the intersection of rod AC and rod BD as a constraint. An optimized structure with freedom limit is designed, as shown in Figure 1(h), which improves the rigidity and safety of the bionic elbow joint [27].

At the same time, this paper studies the problems such as poor comfort in the rehabilitation process and potential side effects in long-term use due to the low matching between the traditional elbow joint structure of the upper limb exoskeleton rehabilitation device and the human elbow joint [28]. By analyzing the kinematics characteristics of human upper limbs, it is determined that in the process of stretching and flexing of human upper limbs, small relative sliding will occur between bones, resulting in that the rotation axis of human elbow joint is not constant. Traditional rotating mechanisms, such as rotating pairs, match the fixed axis with the upper limbs of the human body, resulting in poor rehabilitation comfort of patients and even negative effects. In order to compensate for the change of axis position during the upper limb exoskeleton rehabilitation movement, ensure the synchronization of the man–machine rehabilitation process, and improve the rehabilitation comfort, the elbow joint rotation mechanism in Figure 1(h) is further optimized. Apply the gear–rack mesh structure to the rotation structure to obtain the elbow joint rotation mechanism based on the gear rack, as shown in Figure 1(i). The fixed gear is designed on the rod AC, the rack that meshes with the gear is designed on the rod BD, and the elbow joint rotation mechanism in Figure 1(h) is replaced by the freedom constraint mechanism of the gear rack. During the rotation process of the elbow joint rotation mechanism based on the gear–rack, a small relative displacement is generated between the gear and the rack, which compensates the axis offset of the elbow joint movement.

When the bionic elbow joint rotating mechanism is moving, the gear and rack need to keep meshing all the time. The stability of the rotating mechanism during the rehabilitation process can only be ensured if the distance between the gear axis and the rack plane is fixed. So, this paper designed a locking structure in the rotating mechanism that can move along the sliding plane above the rack, as shown in Figure 1(l).

The upper part of the locking structure is designed with rolling bearings A and B. The locking structure can rotate around the axis of the fixed gear, and the rotary bearing structure C is set below it to keep the distance between the gear and the rack fixed to ensure the meshing state of both. Considering the actual rehabilitation process, the range of motion angles based on the elbow joint is set to 0°−120°. Improve rehabilitation comfort while ensuring rehabilitation safety.

### 2.4. Structure of Forearm and Wrist of the Upper Limb Exoskeleton Rehabilitation Device

This article focuses on the bionic elbow joint, and the following briefly introduces other parts of the upper limb exoskeleton rehabilitation device.

**Figure 2:**
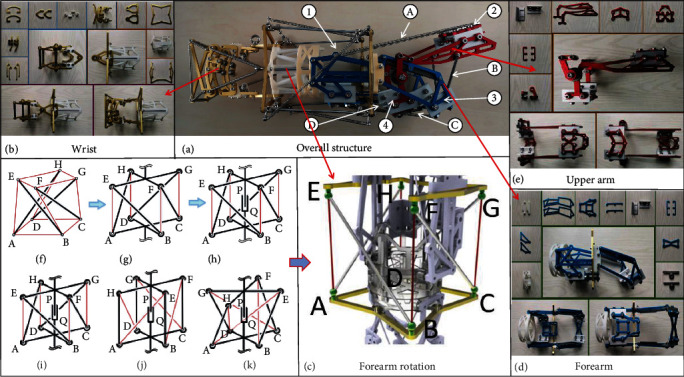
Structure of upper limb exoskeleton rehabilitation device. (a) Overall structure. (b) Wrist structure. (c) Forearm rotation structure. (d) Forearm structure. (e) Upper arm structure. (f–k) Optimization process of forearm rotation structure.

In this paper, the four-bar tensegrity structure is introduced into the structural design of the forearm of the upper limb exoskeleton rehabilitation device. The forearm structure based on the four-bar tensegrity structure is designed with the characteristics of lightweight and self-recovery. The forearm structure was studied with four-bar and 12 cable tensegrity structure. The rotation function of the forearm of the upper limb exoskeleton is realized by using the characteristics of the tensegrity structure, as shown in Figure 2(f). The structure is divided into upper and lower planes ABCD and EFGH, which are, respectively, connected with the wrist and elbow joint. Due to the deformation characteristics of the tensegrity structure, structural optimization is very important.

First of all, in order to ensure that the plane ABCD and plane EFGH will not change during the movement of the forearm structure, the plane ABCD and plane EFGH will be optimized as rigid structures to complete the automatic degree constraint of the plane, as shown in Figure 2(g). Then, in order to avoid the axial displacement of plane ABCD and plane EFGH during rotation, sleeve structures P and Q are designed to constrain the redundant degrees of freedom of the axis to ensure the coaxial rotation between plane ABCD and plane EFGH, as shown in Figure 2(h). By optimizing the sleeve structures P and Q, the constraint of rotation angle was achieved, and the final rotation angle was 135°. Forearm rotation structure is shown in Figure 2(c). Figure 2(i) shows the initial state of forearm rotation, Figure 2(j) shows the state of forearm rotation of 90°, and Figure 2(k) shows the state of maximum rotation angle of 135°.

Analyzing the motion mechanism of human upper limb forearm, the forearm length will change in the process of rotation because the ulna and radius are rigid structures. The forearm structure based on the tensegrity structure will have a length change in the vertical direction during the relative rotation of the plane ABCD and the plane EFGH, which is consistent with the length change of the human forearm, so as to improve the bionic ability and wear comfort. The wrist structure is the most complex bone structure of human upper limbs. By controlling the deformation of driving units such as tendons and ligaments, the wrist joint can realize the flexion (moving toward the palm), extension (moving toward the back of the hand), ulnar deflection (moving toward the little finger), radial deflection (moving toward the thumb), and rotation of the wrist joint.

In this paper, first, complete a structural analysis of the human wrist bones, tendons, and ligaments; then, simplifying the human wrist structure from an institutional perspective and establishing the human wrist skeletal equivalent mechanism. Finally, design a wrist structure with 2 degrees of freedom, as shown in Figure 2(b). Using the mechanical restraint structure, the comfort angles of rehabilitation training were designed as follows: flexion: 60°, extension: 70°, ulnar deflection: 30°, and radial deflection: 20°.

### 2.5. Overall Structure of the Upper Limb Exoskeleton Rehabilitation Device Based on Tensegrity Structure

The overall structure of the upper limb exoskeleton rehabilitation device is shown in Figure 2(a), consisting of four parts: the palm and wrist structure is shown in Figure 2(b), the forearm rotation structure is shown in Figure 2(c), the forearm structure is shown in Figure 2(d), the upper arm structure is shown in Figure 2(e), and the parametric values of the mechanical links are shown in Table 3.

## 3. Control Scheme

According to the principles of modern hemiplegia therapy, the rehabilitation process of upper limbs is mainly divided into three stages: the soft paralysis stage, the spasticity stage, and the recovery stage [29]. Patients have different needs and performance at different stages of rehabilitation. The adaptability and comfort of upper limb rehabilitation exoskeleton directly affect patients' active rehabilitation ability and rehabilitation effect. In view of the low adaptive ability of traditional control schemes, we propose an impedance control scheme of PSO–BP neural network based on fuzzy rehabilitation state evaluator. First, in view of the system stability and security problems caused by the sudden change of impedance such as spasms in the rehabilitation process, we designed a fuzzy rehabilitation state evaluator [30], which uses the fuzzy reasoning method, fully considers the artificial rehabilitation experience, and analyzes the rehabilitation state of patients in real time. Then, we designed a BP neural network-based impedance control scheme for the problem that traditional impedance control cannot adjust impedance parameters in a timely manner according to the changes in the condition of the affected limb [31]. Finally, we optimize the BP neural network for the convergence speed and local optimization seeking problems of the transmission BP neural network, and design a BP neural network impedance parameter controller based on PSO. Improve the self-adaptability ability of the rehabilitation device by optimizing the control scheme.

**Figure 3:**
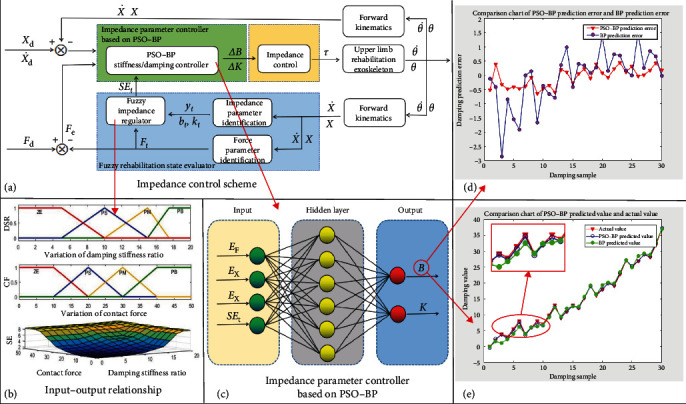
Control scheme. (a) The impedance control scheme of PSO–BP neural network based on fuzzy rehabilitation state evaluator. (b) The input–output relationship of fuzzy rehabilitation state evaluator. (c) The impedance parameter controller based on PSO–BP neural network. (d and e) Simulation result.

The impedance control scheme of PSO–BP neural network based on fuzzy rehabilitation state evaluator is shown in Figure 3(a).

### 3.1. Fuzzy Rehabilitation State Evaluator

During the rehabilitation process, the stiffness and damping of the affected limb will undergo real-time nonlinear changes. So, it is difficult to establish an accurate mathematical model. The fuzzy rehabilitation state evaluator utilizes the advantages of fuzzy control to analyze the influencing parameters such as damping, stiffness [32], and contact force in real time, achieving an effective combination of control strategy and rehabilitation experience. The input variables of the fuzzy rehabilitation state evaluator are the physical status value *y*_*t*_ and contact force *F*_*t*_ of the affected limb, andthe output variable is the rehabilitation status assessment value *SE*_*t*_.The upper limb rehabilitation exoskeleton mass spring damping kinetic model is as follows:

(21) $$\begin{array}{c} f=m\ddot{x}+b\dot{x}+kx. \end{array}$$

*f* is the upper limb and exoskeleton interaction force. *x*, $\dot{x}$,and $\ddot{x}$ are the displacement, velocity, and acceleration of the affected limb, respectively. Considering the rehabilitation process characteristics, the acceleration term $m\ddot{x}$ is neglected as follows:

(22) $$\begin{array}{c} f=b\dot{x}+kx. \end{array}$$

The impedance parameters  *b*,  *k* are calculated using the least squares and sliding average methods, and the impedance parameters are related to the average values of displacement and velocity at each sampling point. The impedance parameters *b*_*t*_ and *k*_*t*_ are identified in real time as follows:

(23) $$\begin{array}{c} \left[\begin{array}{c} b\left(t\right)\\ k\left(t\right) \end{array}\right]={\left[\begin{array}{cc} \sum_{i=t-N+1}^{t}{\dot{x}}^{2}\left(i\right) & \sum_{i=t-N+1}^{t}\dot{x}\left(i\right)x\left(i\right)\\ \sum_{i=t-N+1}^{t}x\left(i\right)\dot{x}\left(i\right) & \sum_{i=t-N+1}^{t}x^{2}\left(i\right) \end{array}\right]}^{-1}\left[\begin{array}{c} \sum_{i=t-N+1}^{t}\dot{x}\left(i\right)f\left(i\right)\\ \sum_{i=t-N+1}^{t}x\left(i\right)f\left(i\right) \end{array}\right]. \end{array}$$

The National Taiwan University utilized a biomechanical model to study the relationship between human–machine interaction, damping, and stiffness in stroke patients [33]. Experimental data show that there are significant differences in human–machine interaction, damping, and stiffness between healthy subjects and stroke patients. The human–machine interaction force, damping, and stiffness have nonlinear characteristics. The increase in stroke severity leads to a significant increase in human–machine interaction and damping values, as well as a slight decrease in stiffness values. Spasm affects the ratio of damping to stiffness, which is consistent with spasm dependent velocity. The damping to stiffness ratio allows a clearer distinction between changes in rehabilitation status. The state evaluator improves the discrimination of the state assessment value *SE*_*t*_ by introducing the damping stiffness ratio *y*_*t*_=(100 × *b*_*t*_)/*k*_*t*_. At the same time, considering the safety risks brought by emergencies in the rehabilitation process, the status evaluator introduces contact force *F*_*t*_ to improve the practicability of the assessment value.

In the fuzzification and defuzzification process, the input variables are divided into four fuzzy subsets (ZE, PS, PM, and PB). The output variables are divided into five fuzzy subsets (ST1, ST2, ST3, ST4, and ST5). The fuzzy inference rules are shown in Table 4.

The input–output relationship of fuzzy rehabilitation state evaluator is shown in Figure 3(b). Input variables: the value range of damping stiffness ratio (DSR) is 0–20, and the value range of contact force (CF) is 0−50 N. The membership functions of DSR and CF are shown in Figure 3(b). Output variable: the value range of rehabilitation status evaluation (SE) value is 0–10. The fuzzy rehabilitation state evaluator divides the rehabilitation state into 10 levels.

Fuzzy control is a control scheme based on fuzzy set theory, fuzzy linguistic variables, and fuzzy logical reasoning. Experiments show that fuzzy control is superior to conventional control in parameter perturbation and external interference for some control systems with unstable parameters. However, fuzzy control also has some shortcomings, such as inability to achieve optimal control, low accuracy caused by large changes in system parameters or external loads. This control strategy embeds fuzzy control into the PSO–BP neural network, effectively improving the overall control accuracy. In the learning process of neural networks, we improve the robustness and adaptability of the overall control scheme by adding 5% random disturbance variables to the input dataset.

### 3.2. Impedance Parameter Controller Based on PSO–BP Neural Network

The impedance control scheme of PSO–BP neural network based on fuzzy rehabilitation state evaluator is shown in Figure 3(a).

Studies have shown that during the rehabilitation process, the affected limb goes through a process from passive training to active training. The rehabilitation exoskeleton can only give full play to the active recovery ability of the limb and improve the rehabilitation effect by changing the rehabilitation parameters in real time. Currently, impedance control is a widely used control strategy in the field of rehabilitation. However, the traditional impedance control cannot adjust the parameters in time according to the changes of the affected limb's condition, and its adaptive ability is low.

In this paper, a impedance parameter controller based on PSO–BP neural network is proposed by making use of the advantages of BP neural network technology such as strong nonlinearity and self-adaptability [34]. At the same time, the PSO algorithm is used to optimize the BP neural network to improve the convergence speed and optimization performance of the BP neural network.

The target impedance control model is as follows [35]:

(24) $$\begin{array}{c} \begin{array}{c} F_{d}-F_{e}=M\left(\ddot{X_{d}}|+|\ddot{X}\right)+B\left(\dot{X_{d}}|-|\dot{X}\right)+K\left(X_{d}-X\right) \end{array}, \end{array}$$

where *M* is the inertia matrix; B is the damping matrix; K is the stiffness matrix, and *X*, $\dot{X}$, and $\ddot{X}$ are the position, velocity, and acceleration quantities, respectively. *X*_d_, $\overset{˙}{X_{d}}$, and $\ddot{X_{d}}$ are the desired position quantity, desired velocity quantity, and desired acceleration quantity, respectively. *F*_d_ is the expected force and *F*_e_ is the actual contact force. Considering the safety of the rehabilitation process, the acceleration of the rehabilitation exercise is low. Neglecting the acceleration term $M\left(\ddot{X_{d}}|-|\ddot{X}\right)$ is as follows:

(25) $$\begin{array}{c} \begin{array}{c} F_{d}-F_{e}=B\left(\dot{X_{d}}|-|\dot{X}\right)+K\left(X_{d}-X\right) \end{array} \end{array}$$

Analyzing the target impedance control model, the impedance parameters *B* and *K* are related to the contact force error *E*_F_=*F*_d_ − *F*_e_, the velocity error $E_{\dot{X}}\dot{=}X_{d}-\dot{X}$, and the position error *E*_X_=*X*_d_ − *X*. Through the self-learning characteristics of BP neural network, the functional relationship between input variables *E*_F_, $E_{\dot{X}}$, and *E*_X_ and output variables *B* and *K* can be deduced.

However, traditional BP neural networks have problems such as long training time and local optimum. Based on the above reasons, we design BP neural network impedance parameter controller based on PSO [36]. The controller takes PSO algorithm as the learning algorithm of BP neural network [37]. By adjusting parameters such as the optimal position of the population, gradually approaching the global optimal solution during the iteration process. It has the advantages of fast convergence and high prediction accuracy.

Considering the influence of rehabilitation state assessment value on impedance control, rehabilitation state assessment value was introduced into the input variable. The impedance parameter controller based on PSO–BP neural network is shown in Figure 3(c). The simulation results of PSO–BP prediction error and BP prediction error are shown in Figure 3(d). The simulation results of PSO–BP predicted value and actual value are shown in Figure 3(e). The simulation results show that compared with the traditional BP neural network impedance control method, this control strategy can effectively reduce the prediction error, improve the prediction accuracy, and improve the self-adaptability of the impedance control scheme. The hyperparameters used in PSO and BP algorithms are shown in Table 5.

## 4. Results

### 4.1. Tensegrity Structure Stability Test

**Figure 4:**
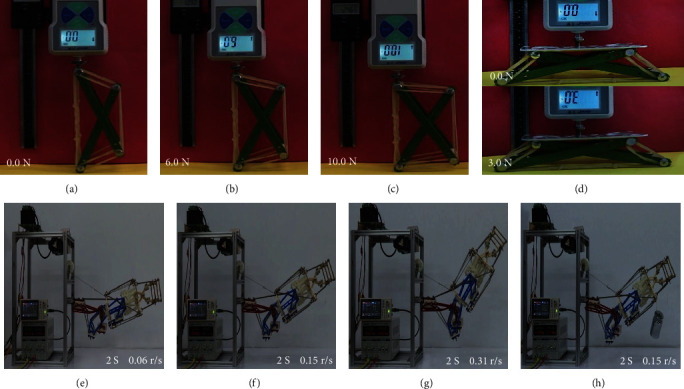
(a–d) Tensegrity structure stability test. (a–c) Horizontal forces. (d) Vertical forces. (e–g) Motion speed test. (h) Load test.

The stability of the tensegrity structure is verified by studying the morphological changes of the basic tensegrity structure units under different pressures. Morphological changes of the tensegrity structure for horizontal forces of 0.0, 6.0, and 10.0 N are shown in Figures 4(a), 4(b), and 4(c), respectively, and for vertical forces of 0.0 and 3.0 N are shown in Figure 4(d). The test results show that the tensegrity structure has good stability in the absence of external forces and good self-recovery and impact resistance in the presence of external forces.

### 4.2. Exoskeleton Motion Speed and Load Test

This test verifies the motion ability and adaptability of the exoskeleton by analyzing the motion state of the exoskeleton under different motion speeds and load conditions. At the same time (2 S), the motion state of exoskeleton at rotation speed of 0.06, 0.15, and 0.31 r/s are shown in Figures 4(e), 4(f), and 4(g), respectively. The motion state of the exoskeleton at a load of 2 kg is shown in Figure 4(h). The test results show that the exoskeleton designed in this paper can realize movement at different speeds and under different loads. The exoskeleton has good movement ability and adaptability.

### 4.3. Rehabilitation Trajectory Test

**Figure 5:**
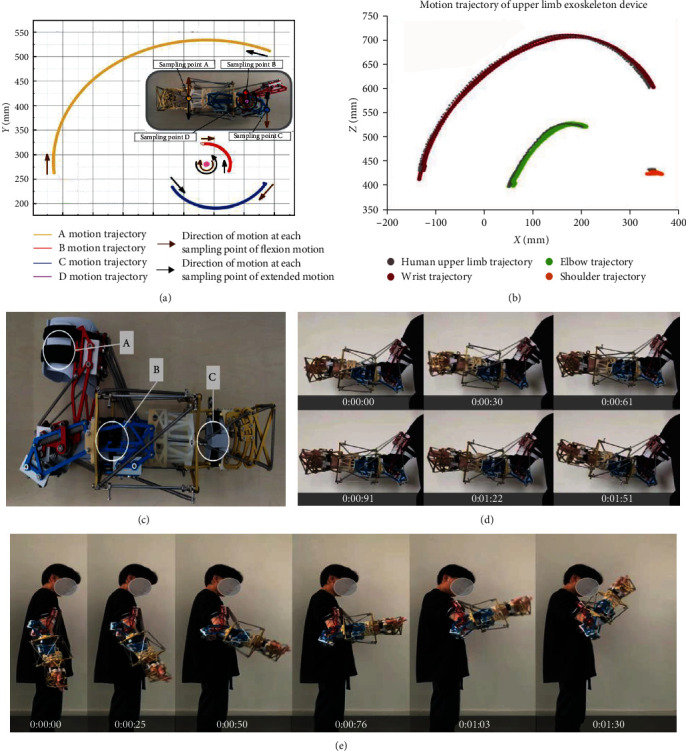
(a and b) Rehabilitation trajectory test. (a) Motion smoothness tests. (b) Motion trajectory test. (c–e) Wearing comfort test. (c) The wearing structure. (d) The export results of pronation and supination. (e) The export results of flexion movement.

Through the rehabilitation trajectory test, the rationality of the upper limb exoskeleton rehabilitation device structure and the smoothness of rehabilitation movement are verified. Four sampling points A, B, C, and D are set on the flexion motion plane as the data capture points of the motion capture camera. In this test, the duration of elbow flexion and extension is set as 60 s, and the sampling frequency is 100 times/s. The motion trajectories of the four sampling points were plotted using the location data of the four sampling points, as shown in Figure 5(a). The test results show that sampling points A, B, and C revolve around sampling point D, and the motion trajectory is a variable diameter smooth curve with sampling point D as the center. We designed six sampling points for the wrist, elbow, and shoulder joints of the upper limb exoskeleton device and the wrist, elbow, and shoulder joints of the human upper limb. During a complete rehabilitation exercise, we use motion capture camera to obtain the motion trajectories of sampling points and then compare the motion trajectories of each joint. The test results show that the motion trajectory of the upper limb exoskeleton device and the human upper limb basically fit, as shown in Figure 5(b).

The above experiments on motion smoothness and trajectory indicate that the structural design of the upper limb exoskeleton rehabilitation device is reasonable and the rehabilitation process is smooth.

### 4.4. Wearing Comfort Test

The images during the flexion, pronation, and supination of the exoskeleton rehabilitation device were collected. Analyze the response and following status of the rehabilitation device, and verify the wearing comfort performance of the rehabilitation device. The wearing comfort performance is verified by the way of human body wearing. The wearing structure is shown in Figure 5(c). A is the upper arm strap, B is the elbow strap, and C is the forearm strap. The tester completed the flexion movement in 1.3 s. According to the motion capture video, the time interval of 26 ms ± 1 ms is selected for single frame export, and the export results are shown in Figure 5(e). The tester completed pronation and supination within 1.5 s, and the time interval was 30 ms ± 1 ms for single frame export. The export results are shown in Figure 5(d). The test results show that the upper limb exoskeleton rehabilitation device does not appear sluggish in the process of flexion, pronation, and supination, shows good dynamic response ability and wear comfort performance.

### 4.5. Flexion Motion Stability Test

**Figure 6:**
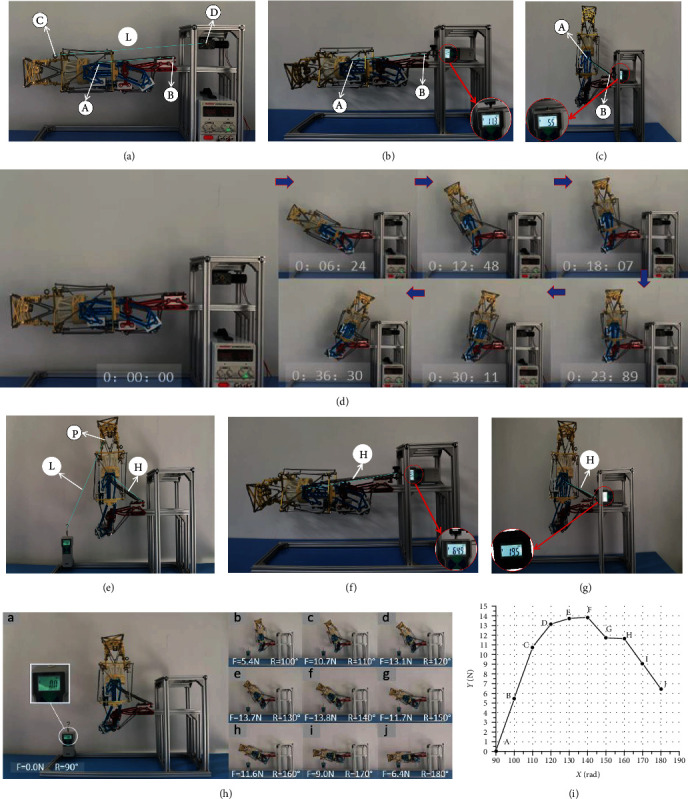
(a–d) Flexion motion stability test. (a) The structure of stability test device. (b) The maximum driving force. (c) The minimum driving force. (d) Capture images of flexion motion. (e–i) Assist ability test. (e) The structure of assist ability test device. (f) The maximum driving force. (g) The minimum driving force. (h) The variation of tension meter reading F and angle R. (i) The angle–tension curve.

By analyzing the process of flexion movement, the stability of rehabilitation movement of the device is verified. C and D are the connection points between the rehabilitation device and the drive motor, and L is the rigid rope that cannot be deformed. A and B are the connection points of the rehabilitation device and the tension detection device, and H is the flexible drive unit. Adjust the flexible drive unit H, and the initial balance state is the upper limb extension state. The minimum driving force is 5.5 N, and the maximum driving force is 11.3 N. The test bench is shown in Figures 6(a), 6(b), and 6(c). Under the action of the driving motor, the rehabilitation device makes a uniform speed flexion motion. The flexion motion pictures of the rehabilitation device were captured with the extended state as the initial state and the time interval of 6 s ± 30 ms, as shown in Figure 6(d). The test results show that the flexion movement angle of the exoskeleton rehabilitation device increases steadily with time, and the rehabilitation movement has good stability and comfort.

### 4.6. Assist Ability Test

Through tensile test, the relationship between assistance and angle during flexion movement is analyzed. Verify the assist ability of the exoskeleton rehabilitation device structure. The test bench is shown in Figures 6(e), 6(f), and 6(g), where P is the fixed point of the distal forearm and L is the nonretractable rigid rope used to connect the tension meter. The elastic driving unit H was adjusted to set the maximum driving force of 64.9 N and the minimum driving force of 19.5 N. During the flexion movement, the variation of the tension meter reading F and the angle R between the forearm and the boom is shown in Figure 6(h), and the angle–tension curve is shown in Figure 6(i). Analyzing the angle–tension curve, the angle between forearm and forearm is in the range of 90°−120°, sampling points a–d show a rapid upward trend, and the power assisting ability increases steadily. When the angle between the forearm and the upper arm ranges from 130° to 150°, sampling points e and f reach the maximum value, and the power assist reaches the maximum value. When the angle is between 150° and 180°, the power assist capacity decreases. The experimental results show that the assisted effect is consistent with the characteristics of upper limb rehabilitation. The upper limb exoskeleton rehabilitation device has a better assisting ability in the rehabilitation space.

## 5. Conclusions

This paper takes the human upper limb as the research object and designs a rehabilitation device and control scheme for the upper limb exoskeleton based on tensegrity structure.

First of all, this paper embarks from the biological characteristics, uses the bionic characteristics of tension structure and designs the bionic elbow based on tension structure. The elbow joint rotation mechanism based on gear and rack is mainly proposed, which compensates the axis offset of elbow joint movement and realizes the relative sliding of human upper limb elbow joint movement. Meanwhile, this paper completes the structural design and structural optimization of the forearm and wrist of the upper limb exoskeleton rehabilitation device.

Then, this paper proposes an impedance parameter control scheme of the PSO–BP neural network based on a fuzzy rehabilitation state evaluator. The simulation results show that the impedance control scheme improves the slow convergence speed and local optimization phenomenon of traditional BP neural network, effectively reduces the prediction error of impedance parameters, and improve the self-adaptability of the upper limb exoskeleton rehabilitation device.

Finally, the test results show that the upper limb exoskeleton rehabilitation device and control strategy proposed in this paper have good wearability, stability, assist ability, and compatibility. It has important application value in the field of medical rehabilitation.

## Figures and Tables

**Table 1:** Coordinates of nodes.

	A	B	C	D
*X*	0	383	245.38	541.92
*Y*	100	100	81.88	161.34

**Table 2:** The vector connection relationship.

Component/relationship	Nodes			
	A	B	C	D
AB	1	−1	0	0
AD	1	0	0	−1
CD	0	0	1	−1
BC	0	1	−1	0
AC	1	0	−1	0
BD	0	1	0	−1

**Table 3:** The parametric values of the mechanical links.

Links	Length	Links	Length
Flexible links A	250 mm	Flexible links B	91 mm
Flexible links C	85 mm	Flexible links D	100 mm
Rigid links 1–3	267 mm	Rigid links 2–4	175.6 mm

**Table 4:** Fuzzy inference rules for rehabilitation status assessment values.

*F*_*t*_	*y*_*t*_			
	ZE	PS	PM	PB
ZE	ST1	ST2	ST3	ST5
PS	ST2	ST3	ST4	ST5
PM	ST3	ST4	ST4	ST5
PB	ST5	ST5	ST5	ST5

**Table 5:** The hyperparameters used in PSO and BP algorithms.

Name	Values	Name	Values
Learning rate	0.1	Acceleration factor	1.49445
Target error	0.001	Minimum performance gradient	0.000006
Momentum factor	0.01	Maximum value of inertia weight	0.9
Population size	30	Minimum value of inertia weight	0.4

## Data Availability

The data that support the findings of this study are available from the corresponding author upon reasonable request.
